# A Novel Method for Dynamically Assessing the Prognosis of Patients with pT1 Gastric Cancer: A Large Population-Based Dynamic Prognostic Analysis

**DOI:** 10.1155/2023/8629166

**Published:** 2023-01-28

**Authors:** Siwei Pan, Mengxuan Cao, Can Hu, Yanqiang Zhang, Yian Du, Zhiyuan Xu, Xiangdong Cheng

**Affiliations:** Department of Gastric Surgery, Institute of Cancer Research and Basic Medical Sciences of Chinese Academy of Sciences, Cancer Hospital of University of Chinese Academy of Sciences, Zhejiang Cancer Hospital, Hangzhou 310022, China

## Abstract

**Background:**

While early gastric cancer (EGC) patients are likely to experience relatively long postoperative survival, certain disease-related findings are associated with a poorer prognosis. This study sought to develop and validate a novel predictive model capable of estimating conditional disease-specific survival (CDSS) in EGC patients.

**Methods:**

A total of 3016 patients diagnosed with pT1NxM0 GC after gastrectomy between 1998 and 2016 were selected from the Surveillance, Epidemiology, and End Results (SEER) database and were separated into training and validation cohorts. Kaplan‒Meier curves and log-rank tests were employed to evaluate DSS, after which univariate and multivariate Cox regression analyses were used to construct a predictive nomogram and to estimate CDSS at 1, 2, and 3 years postoperatively in these patients.

**Results:**

In the training cohort, the 3-year CDSS rose from 89.1% to 94.6% from 0 to 5 years postoperatively, while the 5-year CDSS rose from 84.5% to 92.0%. Cox regression analyses led to the construction of a nomogram that was able to reliably predict 3- and 5-year CDSS at 1, 2, and 3 years postoperatively (all *P* < 0.05) based upon patient age, tumor size, pT stage, pN stage, and the number of retrieved lymph nodes. This model exhibited good discriminative power in the training and validation cohorts (concordance index: 0.791 and 0.813, respectively), and nomogram calibration curves confirmed that actual and predicted survival outcomes were close to one another.

**Conclusions:**

We herein developed a nomogram capable of accurately predicting the CDSS of EGC patients that had survived for multiple years after undergoing surgery.

## 1. Background

Early gastric cancer (EGC) is a subtype of gastric cancer (GC) wherein local tumor invasion extends only into the mucosa (T1a) or submucosa (T1b), whether or not lymph node (LN) metastasis is evident. EGC has a better prognosis following radical resection than do other forms of GC [1–3]. However, patient outcomes vary substantially as a function of individual clinicopathological conditions, and postoperative recurrence rates can be as high as 7.0% [2, 4, 5].

Several prior studies have shown that patients with stage cT1a and cT1b EGC have significantly different prognostic outcomes [6–8]. For example, Kamarajah et al. [6] determined that individuals with stage cT1b disease had significantly higher LN metastasis rates (18% vs. 5%) and a decreased 5-year overall survival (OS) (60% vs. 72%) relative to those with stage cT1a disease following gastrectomy. Significant differences in survival outcomes between EGC patients with and without metastatic LNs have also been reported [7–9]. In prior studies, metastatic LNs have been reported in over 47% of individuals with EGC [10–12]. Age, tumor size, Lauren's classification, and other factors have also been associated with EGC patients' prognosis and postoperative treatment selection [8, 13–16]. For EGC, the favorable prognosis may only apply to patients with pT1aN0M0 GC, while for the other types of EGC, maintaining the traditional perception may have a negative impact on clinical decision-making and the formulation of follow-up schemes. Therefore, an accurate, evidence-based tool be developed to individualize and dynamically assess the prognosis of EGC is more conducive to promoting the individualized treatment.

Conditional survival (CS) or conditional disease-specific survival (CDSS) is a prognostic indicator that is utilized for dynamic evaluation of patient prognosis, allowing clinicians to more accurately gauge patient prognosis in light of the fact that the risk of death declines as survival time increases. For example, if a patient with advanced disease has survived 5 years after surgery, we generally consider that the probability of survival of another 3 years, i.e., cumulative survival of 8 years, will be optimistic due to the declines of the risk of death, rather than the almost zero 8-year survival rate that we normally recognize [1, 17, 18]. CS has been confirmed to offer valuable prognostic information when used for the postoperative surveillance of cancer patients, making it a promising tool for patient management and treatment selection [19–22].

In the present study, we therefore sought to develop and validate a nomogram capable of predicting EGC patients' CDSS based on data derived from the Surveillance, Epidemiology, and End Results (SEER) database.

## 2. Materials and Methods

### 2.1. Study Population

The SEER data browser was used to access all information in the SEER database, which compiled incidence and survival data pertaining to roughly 28% of the US population [23, 24]. The SEER-stat software (SEER^*∗*^Stat 8.3.6) was used to screen the cohort data in the present study. Patients eligible for inclusion in the present study were those in the SEER database who had undergone gastrectomy and been diagnosed with gastric adenocarcinoma from 1998 to 2016 exhibiting tumor invasion of the mucosa (T1a) or submucosa (T1b) without distant metastasis. Patients were excluded from the study if they met the following criteria: (1) patients with tumors at the cardia; (2) patients <18 or >90 years old; (3) patients without clear clinical or follow-up information; (4) patients that survived for <1 month; (5) patients who died as a consequence of diseases other than GC. Using these criteria, 3016 eligible patients were identified for further analysis.

Data extracted for patients included in the present study included patient sex, age, race, primary tumor location, primary tumor size, grade, pT and pN stage, the number of retrieved and metastatic LNs, information regarding patient adjuvant therapy, follow-up duration, and patient survival status as of most recent follow-up (Nov. 2018). The depth of invasion and LN metastasis were defined as per the 8^th^ edition of the American Joint Committee on Cancer (AJCC) Cancer Staging Manual [25].

### 2.2. Nomogram Development and Validation

Patients were randomly assigned to training and validation cohorts (*n* = 2011 and *n* = 1005, respectively) at a 2 : 1 ratio. A Cox proportional hazard regression model was then employed to identify predictors of disease-specific survival (DSS) in these patient cohorts. Variables evaluated using this model included sex, age, race, primary tumor location, primary tumor size, grade, pT and pN stage, the number of retrieved and metastatic LNs, and patient adjuvant therapy information. The results of this analysis were used to construct a nomogram capable of predicting EGC patient DSS and CDSS. The advantage of the nomogram is to quantify variables into points specifically, so that users can more conveniently obtain the total point of each patient according to their clinical information and then find the corresponding DSS and CDSS. When using the nomogram for external validation, the prognosis of patients can be easily predicted after obtaining clinical information and the total points of patients.

Nomogram performance was assessed based upon discrimination and calibration criteria. Harrell's concordance index (C-index) was used to quantify the discriminative power of the model, with higher C-index values corresponding to greater model accuracy [26, 27]. A C-index value > 0.75 is generally consistent with good model discrimination. Calibration curves were used to compare actual patient survival to that predicted using our constructed nomogram, with a bootstrapping method being utilized to decrease the potential for bias [28]. For validation, this nomogram was used to calculate scores for each patient in the validation cohort. A decision curve analysis (DCA) was then performed to measure the clinical utility of this model by measuring the net benefits for a group of threshold probabilities [29].

### 2.3. Statistical Analysis

DSS was defined as the time between tumor resection and death due to GC. As mortality risk changes dynamically over time after gastrectomy, we used CDSS as a metric for evaluating DSS at specific time points using the following formula: CDSS (*y | x*) = DSS(*x* + *y*)/DSS(*x*), where DSS(*x*) corresponds to the actual DSS at time point *x*, and *y* corresponds to the additional expected survival duration after time point *x* [17, 30]. For example, if a given patient has survived for one year postsurgery, their probability of surviving an additional 3 years can be calculated as follows: CDSS (3 | 1) = DSS(4)/DSS(1). DSS was calculated using Kaplan–Meier curves, with log-rank tests being used for statistical verification. Univariate and multivariate Cox proportional hazards regression models were used to identify predictors of patient prognosis, while follow-up was quantified via the reverse Kaplan–Meier method [31, 32].

R software (v 3.5.3; R Foundation for Statistical Computing, Vienna, Austria) and SPSS (v 23.0; SPSS Inc., IL, USA) were used to conduct all statistical testing, with a two-tailed*P* < 0.05 as the significance threshold for this study. For the R software, the *muhaz* package was used to dynamically analyze the hazard ratio (HR) of patients after surgery. The *survival* package was used to analyze the prognosis of patients and calculate the c-index. The *rms*, *foreign*, and *nomogramFormula* packages were used to develop the nomogram and verify the validity of the model. The *rmda* package was used to perform DCA. For the SPSS, we mainly used this to perform univariate and multivariate Cox proportional hazards regression models and identify predictors to develop the nomogram.

## 3. Results

### 3.1. Patient Clinicopathological Characteristics

**Figure 1:**
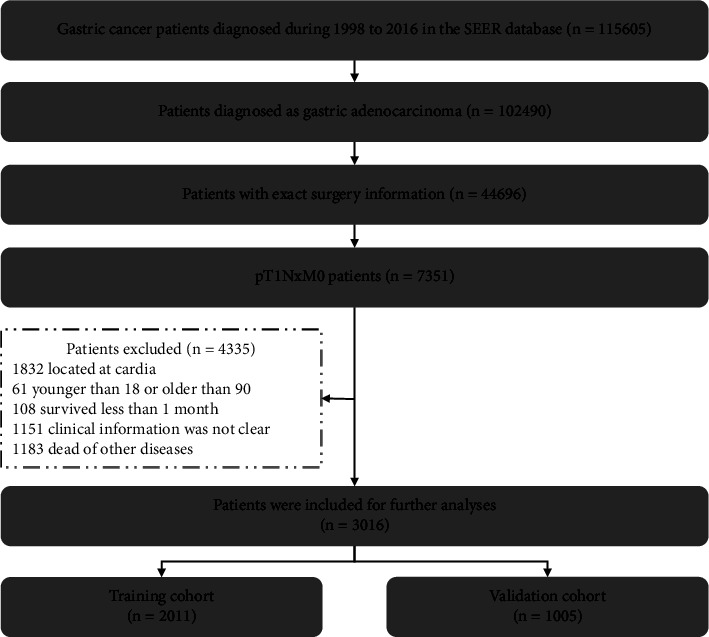
Patients' screening process for the present study from the SEER database.

In total, 3016 EGC patients were incorporated into the present analyses (Figure 1). The clinicopathological characteristics of the overall, training, and validation patient cohorts in this study are shown in Table 1. More than half of patients in the present study were male. The overall patient cohort had a median age of 69 years (IQR: 59–76), and over 60% (*n* = 1868) of patients were diagnosed with stage pT1b disease. The mean number of LNs retrieved per patient was 16.3 ± 14.0, with > 15 LNs being obtained for 1256 (41.64%) patients. LN metastasis was detected in 20.52% of these patients. Follow-up times ranged from 1 to 226 months, and no patients were lost to follow-up. The median patient follow-up time was calculated as being 76 months.

### 3.2. Study Cohort Survival Analyses

**Figure 2:**
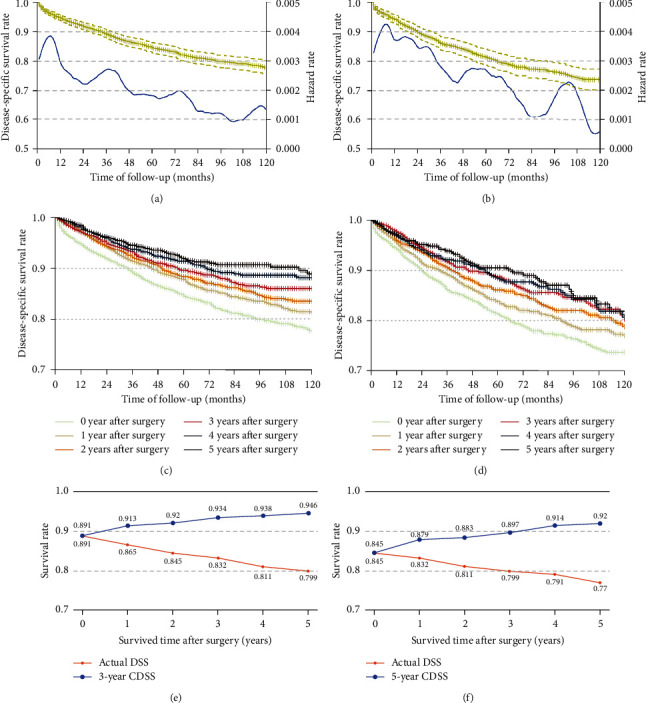
Survival analyses of the study cohorts. Kaplan‒Meier DSS curves (blue line) and HR curves (yellow line) of the training cohort (a) and validation cohort (b); Kaplan‒Meier DSS curves, started from different years after surgery, of the training (c) and validation (d) cohorts; actual DSS and 3-year (e) and 5-year (f) CDSS are compared for EGC patients survived for multiple years after surgery in the training cohort.

In our overall study cohort, the 3- and 5-year DSS rates were 88.1% and 83.4%, respectively. HR curves were generated for these patients after their random assignment to training and validation cohorts (Figure 2), which had respective median follow-up times of 76 and 75 months. The 3- and 5-year DSS rates were 89.1% and 84.5%, respectively, in the training cohort, and 86.0% and 81.3% in the validation cohort. HR curves for both cohorts confirmed that the risk of mortality was highest within the first year after surgery, with this risk declining thereafter (Figures 2(a) and 2(b)). Survival analyses similarly confirmed that the extension of postoperative survival time increased the odds of patients surviving for additional time (Figures 2(c) and 2(d)). As such, actual DSS does not reliably reflect the prognosis of EGC patients who have survived for multiple years after gastrectomy, indicating that CDSS is a more reliable index for evaluating these individuals. The actual DSS, as well as the 3- and 5-year CDSS of patients in the training cohort who had survived 0–5 years after surgery are shown in Figures 2(e) and 2(f). The 3-year CDSS at 1-year postsurgery was 91.3%, and it rose to 94.6% for patients that had survived 5 years postoperatively. In contrast, actual 4- and 8-year DSS rates postsurgery were just 86.5% and 79.9%, respectively. Comparable results were also obtained when assessing 5-year CDSS and actual DSS.

### 3.3. Identification of Predictors of EGC Patient DSS and CDSS

To develop a nomogram capable of predicting EGC patient DSS and CDSS, we next utilized Cox proportional hazards regression models to identify independent predictors of these outcomes (Tables 2 and 3). Univariate analyses of the training cohort revealed age, tumor size, pT and pN stage, the number of retrieved LNs, and adjuvant therapy to all be associated with patient prognosis (all *P* < 0.05). These factors were incorporated into a subsequent multivariate analysis, which identified age, tumor size, pT and pN stage, and the number of retrieved LNs to be independent predictors of patient survival (all *P* < 0.05).

### 3.4. Development and Validation of a Nomogram for Predicting DSS and CDSS

**Figure 3:**
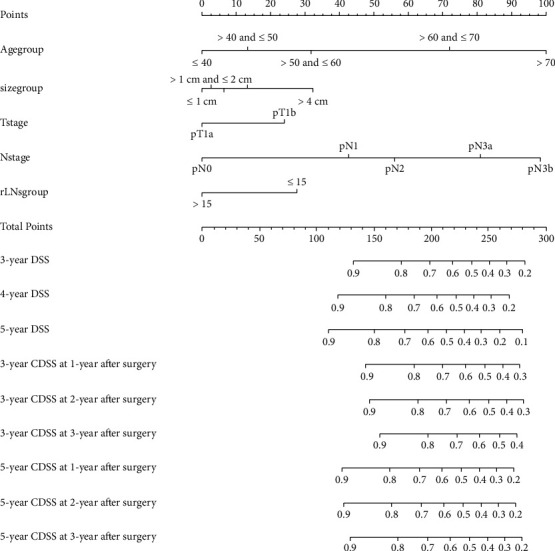
The nomogram for dynamically predicting the prognosis of EGC patients who underwent surgery.

**Figure 4:**
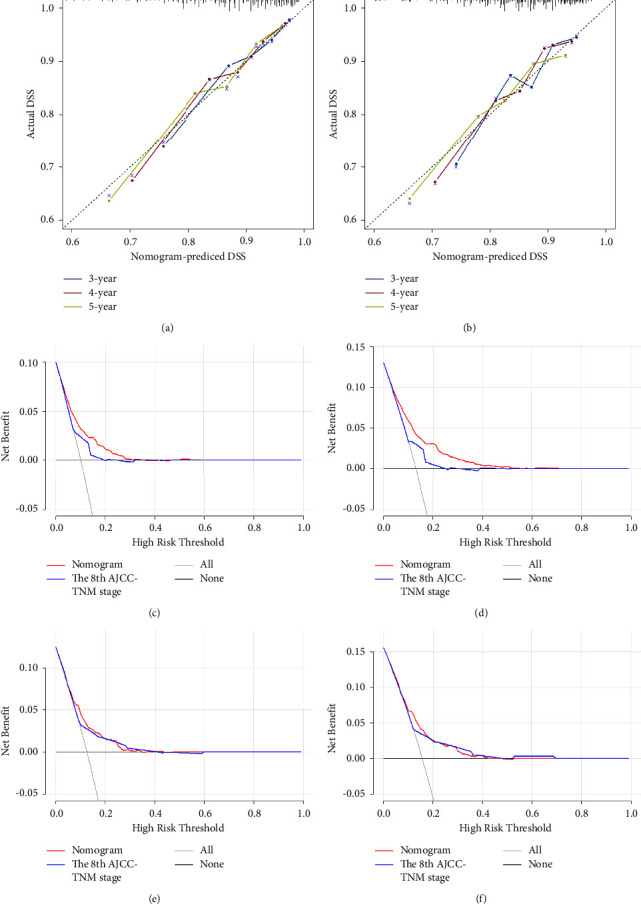
The validity and advantage comparison of the nomogram. Calibration curves of the nomogram in the training cohort (a) and validation cohort (b). DCA curves, analyzed via DSS, of the nomogram compared with the 8^th^ TNM staging system: (c) 3 years and (d) 5 years after surgery in the training cohort; (e) 3 years and (f) 5 years after surgery in the validation cohort.

We next used the results of the above multivariate analysis to construct a nomogram capable of predicting EGC patient 3-, 4-, 5-year DSS, as well as 3- and 5-year CDSS at 1, 2, or 3 years after surgery in the training cohort (Figure 3). The resultant nomogram enables users to calculate an individualized risk score that can estimate patient-specific DSS and CDSS. The C-index value of this nomogram was 0.791 (95% confidence interval (CI): 0.767–0.815) in the training cohort, and similar discrimination ability was observed in the validation cohort in which the C-index value was 0.813 (95% CI: 0.778–0.848). In contrast, the C-index values of AJCC-TNM staging system in the training and validation cohorts were only 0.601 (95% CI: 0.587–0.615) and 0.597 (95% CI: 0.580–0.614), respectively. Calibration curves were additionally constructed to compare predicted and actual survival rates in both patient cohorts (Figures 4(a) and 4(b)), revealing that this nomogram could effectively estimate EGC patient prognosis under all tested conditions without any significant error. DCA curves, analyzed via DSS, additionally revealed that the clinical utility of this nomogram was promising and showed a better clinical utility to predict the death of patients than the 8^th^ TNM staging system at different points after surgery (Figures 4(c)–4(f)).

## 4. Discussion

Advances in diagnostic and therapeutic technologies are steadily improving GC patient survival rates, with D2 lymphadenectomy with gastrectomy and continuous postoperative treatment being particularly beneficial in this regard [33]. As the number of GC survivors continues to rise, particularly among those with EGC, it is increasingly important that tools be developed to dynamically evaluate patient prognosis so that postoperative treatment can be individually tailored. Herein, we developed and validated a nomogram that was able to predict EGC patient CDSS and DSS at 3, 4, and 5 years postoperatively while also enabling the reliable prediction of additional 3- or 5-year survival after having survived for a given number of years after gastrectomy.

EGC patients are generally considered to have better survival rates than other GC patients, with individuals with EGC that do not exhibit mLNs having a 5-year OS of approximately 90% [1–3]. However, EGC is a heterogeneous condition, with factors such as LN metastasis and tumor stage being closely related to patient outcomes [6, 7, 9, 16]. Lee et al. [9] found that the 5-year OS of individuals with T1N0, T1N1, T1N2, and T1N3 disease was 99.3%, 96.8%, 72.7%, and 0.0%, respectively (*P* < 0.001). Yang et al. [16] found that metastatic LNs were present in just 2.4% of individuals with T1a stage disease, whereas this incidence rose to 11.0% in those with T1b stage disease. Tumor size and metastatic LN incidence were also confirmed to be significantly related to one another (*P* < 0.05), and the 5-year DSS rates of T1a and T1b stage patients in their study were 90.6% and 81.4%, respectively. Consistent with these results, Kamarajah et al. [6] found that EGC patients' prognoses varied as a function of patient clinicopathological characteristics. Evaluating patient OS based solely on tumor stage is thus not sufficient to reliably identify EGC patients likely to experience favorable or unfavorable outcomes. OS or DSS rates calculated in most studies also focus on a single defined time point, and thus fail to reliably reflect dynamic changes in postoperative prognosis, potentially leading to an inaccurate understanding of disease status and associated risk.

Herein, we incorporated the concept of CS as a means of accounting for dynamic changes in patient mortality risk over time after surgery, as this approach is particularly valuable when evaluating patients that have experienced long-term survival [21]. Given the prolonged survival of most EGC patients, we posited that CS or CDSS would be a more reliable index for the assessment of these patients. Wang et al. [17] determined that patients with unfavorable disease characteristics exhibited larger increases in CS, potentially providing some degree of psychological comfort to these individuals. Another study examined postoperative changes in CS under different surveillance strategies, enabling the authors to propose a means of optimizing the National Comprehensive Cancer Network and Japanese Gastric Cancer Association treatment guidelines [34, 35]. When CS is >95.0%, researchers have suggested that patient follow-up frequency can be reduced to avoid excess patient re-evaluation [36], as the cancer-related mortality risk for these patients was similar to that of the general population. We similarly observed gradual increases in 3- and 5-year CDSS in the study population as postoperative survival time increased. At 8 years postsurgery, actual DSS in our patient cohort was just 79.9%, while our model revealed a CDSS for 3 additional years of up to 94.6% for patients that had survived 5 years postsurgery. Similarly, while the actual 10-year DSS for these patients was just 77.0%, the 5-year CDSS for patients that had already survived 5 years postsurgery was 15.0% higher than the overall DSS for this 10-year endpoint. We also found that CDSS rose as postoperative survival time grew longer, potentially providing insights that may guide clinicians in the formulation of appropriate treatment and surveillance strategies. Individualized CS or CDSS-based analyses such as the nomogram developed herein also have the potential to improve patient psychological comfort and to reduce associated postoperative costs.

Chen et al. [37] first reported a nomogram capable of predicting the cancer-specific survival (CSS) and the conditional probability for their multicenter cancer patient cohorts. In that study, the authors incorporated age, tumor site, tumor size, depth of invasion, number of examined LNs, number of metastatic LNs, and surgical margin into their final nomogram. However, their model may not be applicable to GC patients at a particular disease stage given that the study population included all GC patients. In this study, we therefore sought to facilitate precision medicine analyses by specifically evaluating EGC patients in order to identify independent predictors of CDSS and DSS for this particular population.

There are a number of limitations to this analysis. For one, this study was reliant upon retrospective data such that selection bias cannot be excluded, although the selection criteria for this study were based upon the long-time horizon of the SEER database. Second, adjuvant therapy has been repeatedly shown to be relevant to patient prognosis, yet the information in the SEER database regarding patient adjuvant treatment was often unclear, potentially biasing our findings. Third, we did not validate our nomogram on an external patient cohort separate from the SEER database. In future studies, we will include additional patient information and will perform prospective clinical studies aimed at validating and further optimizing our nomogram.

## 5. Conclusions

In conclusion, the nomogram that we developed and validated in this study was able to reliably predict EGC patient CDSS. As CDSS can guide patient postoperative management and follow-up frequency, this tool may be valuable in clinical settings and has the potential to alleviate mental and financial stress for patients found to have higher odds of survival. The applicability of this nomogram as a tool for evaluating EGC patients from other institutions will, however, require future validation.

## Figures and Tables

**Table 1:** Demographic and pathological characteristics of patients selected for further analyses from the SEER database.

Characteristics	Whole cohort		Training cohort		Validation cohort	
	*N*	(%)	*n*	(%)	*n*	(%)
*Sex*						
Male	1628	53.98	1064	52.91	564	56.12
Female	1388	46.02	947	47.09	441	43.88

*Age*						
≤40	95	3.15	61	3.03	34	3.38
>40 and ≤50	242	8.02	159	7.91	83	8.26
>50 and ≤60	502	16.64	340	16.91	162	16.12
>60 and ≤70	829	27.49	557	27.70	272	27.06
>70	1348	44.69	894	44.46	454	45.17

*Race*						
White	1514	50.20	1015	50.47	499	49.65
Black	416	13.79	278	13.82	138	13.73
Others	1086	36.01	718	35.70	368	36.62

*Tumor location*						
Upper	112	3.71	71	3.53	41	4.08
Middle	454	15.05	289	14.37	165	16.42
Lower	1355	44.93	905	45.00	450	44.78
Overlapping	1095	36.31	746	37.10	349	34.73

*Grade*						
Well differentiated	371	12.30	249	12.38	122	12.14
Moderately differentiated	956	31.70	644	32.02	312	31.04
Poorly differentiated	1629	54.01	1077	53.56	552	54.93
Undifferentiated	60	1.99	41	2.04	19	1.89

*Size*						
≤1 cm	705	23.38	464	23.07	241	23.98
>1 cm and ≤2 cm	936	31.03	625	31.08	311	30.95
>2 cm and ≤3 cm	606	20.09	400	19.89	206	20.50
>3 cm and ≤4 cm	352	11.67	240	11.93	112	11.14
>4 cm	417	13.83	282	14.02	135	13.43

*pT stage*						
pT1a	1148	38.06	781	38.84	367	36.52
pT1b	1868	61.94	1230	61.16	638	63.48

*pN stage*						
pN0	2397	79.48	1585	78.82	812	80.80
pN1	367	12.17	261	12.98	106	10.55
pN2	180	5.97	120	5.97	60	5.97
pN3a	59	1.96	35	1.74	24	2.39
pN3b	13	0.43	10	0.50	3	0.30

*Number of retrieved LNs*						
≤15	1760	58.36	1178	58.58	582	57.91
>15	1256	41.64	833	41.42	423	42.09

*Adjuvant therapy*						
Yes	499	16.55	333	16.56	166	16.52
No/unknown	2517	83.45	1678	83.44	839	83.48

*n*, number of patients; LNs, lymph nodes.

**Table 2:** Univariate Cox hazards regression analyses of independent prognosis factors for the prediction nomogram.

Characteristics	Univariate analysis		
	HR	95% CI	*P* value
Sex			0.174
Male	Ref		
Female	0.860	0.692–1.069	0.174
Age			**<0.001**
≤40	Ref		
>40 and ≤50	1.314	0.362–4.776	0.678
>50 and ≤60	1.731	0.525–5.705	0.367
>60 and ≤70	3.503	1.108–11.079	0.033
>70	6.060	1.938–18.953	0.002
Size			**<0.001**
≤1 cm	Ref		
>1 cm and ≤2 cm	1.237	0.872–1.754	0.233
>2 cm and ≤3 cm	1.780	1.247–2.542	0.001
>3 cm and ≤4 cm	1.941	1.325–2.842	<0.001
>4 cm	2.461	1.693–3.577	0.001
Grade			0.299
Well differentiated	Ref		
Moderately differentiated	1.470	0.983–2.197	0.060
Poorly differentiated	1.306	0.887–1.923	0.176
Undifferentiated	1.248	0.519–2.999	0.620
Tumor location			0.270
Upper	Ref		
Middle	0.755	0.423–1.348	0.342
Lower	0.629	0.369–1.073	0.089
Overlapping	0.728	0.427–1.242	0.244
pT stage			**<0.001**
pT1a	Ref		
pT1b	1.987	1.548–2.551	**<0.001**
pN stage			**<0.001**
pN0	Ref		
pN1	2.427	1.857–3.172	<0.001
pN2	2.562	1.812–3.624	<0.001
pN3a	2.564	1.359–4.839	0.004
pN3b	5.485	2.257–13.330	<0.001
Number of retrieved LNs			**<0.001**
≤15	Ref		
>15	0.651	0.514–0.824	<0.001
Adjuvant therapy			**0.001**
Yes	Ref		
No/Unknown	1.521	1.175–1.968	0.001

HR, hazard rate; CI, confidence interval. Bold values indicate the significant difference with *P* < 0.05.

**Table 3:** Multivariate Cox hazards regression analyses of independent prognosis factors for the prediction nomogram.

Characteristics	Multivariate analysis			
	*β*-coefficients	HR	95% CI	*P* value
Age				**<0.001**
≤40	0	Ref		
>40 and ≤50	0.259	1.296	0.356–4.720	0.694
>50 and ≤60	0.589	1.802	0.546–5.951	0.334
>60 and ≤70	1.312	3.715	1.170–11.794	0.026
>70	1.809	6.104	1.943–19.174	0.002
Size				**0.014**
≤1 cm	0	Ref		
>1 cm and ≤2 cm	0.053	1.054	0.740–1.501	0.770
>2 cm and ≤3 cm	0.237	1.268	0.879–1.828	0.204
>3 cm and ≤4 cm	0.574	1.776	1.211–2.603	0.003
>4 cm	0.126	1.134	0.756–1.701	0.542
pT stage				**0.001**
pT1a	0	Ref		
pT1b	0.441	1.554	1.198–2.016	0.001
pN stage				**<0.001**
pN0	0	Ref		
pN1	0.838	2.311	1.697–3.147	<0.001
pN2	1.100	3.003	2.001–4.506	<0.001
pN3a	1.577	4.838	2.384–9.818	<0.001
pN3b	1.857	6.407	2.469–16.626	<0.001
Number of retrieved LNs				**<0.001**
≤15	0	Ref		
>15	−0.501	0.606	0.473–0.776	<0.001
Adjuvant therapy				0.367
Yes		Ref		
No/unknown		1.164	0.837–1.619	0.367

HR, hazard rate; CI, confidence interval. Bold values indicate the significant difference with *P* < 0.05.

## Data Availability

The datasets generated and analyzed during the current study are available in the SEER database (https://seer.cancer.gov/) and from the corresponding authors upon reasonable request.

## Abbreviations

EGC: Early gastric cancer
GC: Gastric cancer
LN: Lymph node
OS: Overall survival
CS: Conditional survival
CDSS: Conditional disease-specific survival
SEER: Surveillance, Epidemiology, and End Results
AJCC: American Joint Committee on Cancer
DSS: Disease-specific survival
DCA: Decision curve analysis
HR: Hazard ratio
CI: Confidence interval
CSS: Cancer-specific survival.
